# Supplementary material for: Using hyperspectral leaf reflectance to estimate photosynthetic capacity and nitrogen content across eastern cottonwood and hybrid poplar taxa
Source: PLoS One. 2022 Mar 10;17(3):e0264780. doi: 10.1371/journal.pone.0264780 (PMC8912144; doi:10.1371/journal.pone.0264780)
Supplement: S1 Fig — D×T and T×D taxa were combined and shown as D×T, and (D×N)×M taxa was included in D×N (S1 Table). D×D = P. deltoides × P. deltoides, D×M = P. deltoides × P. maximowiczii, D×N = P. deltoides × P. nigra, (D×N)×M = P. deltoides × P. nigra × P. maximowiczii, D×T = P. deltoides × P. trichocarpa, T×D = P. trichocarpa × P. deltoides, and T×M = P. trichocarpa × P. maximowiczii. (DOCX) [file pone.0264780.s001.docx]

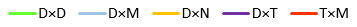


**S1 Fig. Locations of wavelengths selected by the LASSO models for estimating *V*_cmax_, *J*_max_, TPU, and *N*_area_.** D×T and T×D taxa were combined and shown as D×T, and (D×N)×M taxa was included in D×N (S1 Table). D×D = *P*. *deltoides* × *P*. *deltoides*, D×M = *P*. *deltoides* × *P*. *maximowiczii*, D×N = *P*. *deltoides* × *P*. *nigra*, (D×N)×M = *P*. *deltoides* × *P*. *nigra* × *P*. *maximowiczii*, D×T = *P*. *deltoides* × *P*. *trichocarpa*, T×D = *P*. *trichocarpa* × *P*. *deltoides*, and T×M = *P*. *trichocarpa* × *P*. *maximowiczii*.
