# Supplementary material for: Using hyperspectral leaf reflectance to estimate photosynthetic capacity and nitrogen content across eastern cottonwood and hybrid poplar taxa
Source: PLoS One. 2022 Mar 10;17(3):e0264780. doi: 10.1371/journal.pone.0264780 (PMC8912144; doi:10.1371/journal.pone.0264780)
Supplement: S1 Table — Monroe and Pontotoc columns describe the number of leaves on which measurements were made and measurement dates. There were a total of seven taxa and 62 genotypes measured at both study sites. (DOCX) [file pone.0264780.s002.docx]

**S1 Table. List of taxa and genotypes measured at the Monroe and Pontotoc sites.** Monroe and Pontotoc columns describe the number of leaves on which measurements were made and measurement dates. There were a total of seven taxa and 62 genotypes measured at both study sites.

| **No.** | **Taxa** | **Genotype** | **Monroe** | |  | **Pontotoc** | |
| --- | --- | --- | --- | --- | --- | --- | --- |
|  |  |  | **No. of leaves** | **Measurement dates** |  | **No. of leaves** | **Measurement dates** |
| 1. | *Populus deltoides* × *Populus deltoides* (D×D) | ST66 |  |  |  | 1 | 7/15/2020 |
|  |  | ST70 |  |  |  | 1 | 7/15/2020 |
|  |  | ST75 | 4 | 7/19/2019 (2 leaves) and 9/4/2019 (2 leaves) |  | 1 | 7/15/2020 |
|  |  | S7C2 |  |  |  | 1 | 7/14/2020 |
|  |  | S7C4 |  |  |  | 1 | 7/16/2020 |
|  |  | 106B-1 | 2 | 7/19/2019 (2 leaves) |  |  |  |
|  |  | 3-1 |  |  |  | 1 | 7/16/2020 |
|  |  | 6-1 |  |  |  | 1 | 7/15/2020 |
|  |  | 6-4 | 2 | 7/25/2019 (2 leaves) |  |  |  |
|  |  | 6-5 | 2 | 7/25/2019 (2 leaves) |  | 1 | 7/15/2020 |
|  |  | 47-5 | 1 | 7/25/2019 |  |  |  |
|  |  | 120-4 |  |  |  | 1 | 7/14/2020 |
|  |  | 19 |  |  |  | 1 | 7/15/2020 |
|  |  | 22 |  |  |  | 1 | 7/14/2020 |
|  |  | 110412 |  |  |  | 1 | 7/16/2020 |
|  |  | 112107 | 4 | 7/25/2019 (2 leaves) and 9/4/2019 (2 leaves) |  |  |  |
| 2. | *Populus deltoides* × *Populus maximowiczii* (D×M) | 6323 |  |  |  | 1 | 7/16/2020 |
|  |  | 6329 |  |  |  | 1 | 7/16/2020 |
|  |  | 8015 | 2 | 7/26/2019 (2 leaves) |  |  |  |
|  |  | 8019 |  |  |  | 1 | 7/16/2020 |
|  |  | 9225 | 2 | 7/26/2019 (2 leaves) |  |  |  |
|  |  | 9707 |  |  |  | 1 | 7/16/2020 |
|  |  | 9711 | 2 | 7/26/2019 (2 leaves) |  |  |  |
|  |  | 9225 |  |  |  | 1 | 7/14/2020 |
|  |  | 11690 |  |  |  | 1 | 7/16/2020 |
|  |  | 13693 |  |  |  | 1 | 7/16/2020 |
|  |  | 13724 | 2 | 7/26/2019 (2 leaves) |  | 1 | 7/15/2020 |
|  |  | 14492 | 4 | 7/26/2019 (2 leaves) and 9/4/2019 (2 leaves) |  |  |  |
|  |  | 24033 |  |  |  | 1 | 7/14/2020 |
|  |  | 24056 |  |  |  | 1 | 7/14/2020 |
|  |  | 24066 |  |  |  | 1 | 7/15/2020 |
|  |  | 24114 |  |  |  | 1 | 7/14/2020 |
|  |  | 24120 |  |  |  | 1 | 7/14/2020 |
|  |  | 24159 |  |  |  | 1 | 7/16/2020 |
|  |  | 29310 |  |  |  | 1 | 7/16/2020 |
| 3. | *Populus deltoides* × *Populus nigra* (D×N) | 433 | 4 | 7/18/2019 (2 leaves) and 9/2/2019 (2 leaves) |  | 1 | 7/14/2020 |
|  |  | 11785 |  |  |  | 1 | 7/15/2020 |
|  |  | 11789 |  |  |  | 1 | 7/15/2020 |
|  |  | 11795 |  |  |  | 1 | 7/16/2020 |
|  |  | 11797 |  |  |  | 1 | 7/15/2020 |
|  |  | 11802 |  |  |  | 1 | 7/16/2020 |
|  |  | 11822 | 4 | 7/18/2019 (2 leaves) and 9/2/2019 (2 leaves) |  |  |  |
|  |  | 11840 | 4 | 7/18/2019 (2 leaves) and 9/2/2019 (2 leaves) |  | 1 | 7/15/2020 |
|  |  | 11859 | 4 | 7/18/2019 (2 leaves) and 9/2/2019 (2 leaves) |  |  |  |
|  |  | 11867 | 4 | 7/18/2019 (2 leaves) and 9/2/2019 (2 leaves) |  |  |  |
|  |  | 13849 |  |  |  | 1 | 7/15/2020 |
|  |  | 14278 |  |  |  | 1 | 7/15/2020 |
|  |  | 14340 |  |  |  | 1 | 7/15/2020 |
| 4. | *Populus deltoides* × *Populus nigra* × *Populus maximowiczii* ((D×N)×M) | 24250 |  |  |  | 1 | 7/15/2020 |
| 5. | *Populus deltoides* × *Populus trichocarpa* (D×T) | 7903 | 2 | 7/25/2019 (2 leaves) |  | 1 | 7/14/2020 |
|  |  | 7938 | 2 | 7/25/2019 (2 leaves) |  |  |  |
|  |  | 8717 | 2 | 7/25/2019 (2 leaves) |  |  |  |
|  |  | 8729 | 2 | 7/26/2019 (2 leaves) |  | 1 | 7/14/2020 |
|  |  | 10016 |  |  |  | 1 | 7/14/2020 |
| 6. | *Populus trichocarpa* ×*Populus deltoides* (T×D) | 9755 | 2 | 7/25/2019 (1 leaf) and 7/26/2019 (1 leaf) |  |  |  |
| 7. | *Populus trichocarpa* × *Populus maximowiczii* (T×M) | 24245 |  |  |  | 1 | 7/16/2020 |
|  |  | 24301 |  |  |  | 1 | 7/14/2020 |
|  |  | 24304 |  |  |  | 1 | 7/14/2020 |
|  |  | 24326 |  |  |  | 1 | 7/16/2020 |
|  |  | 24340 |  |  |  | 1 | 7/14/2020 |
|  |  | 29262 |  |  |  | 1 | 7/14/2020 |
|  |  | 29270 |  |  |  | 1 | 7/16/2020 |
|  | **TOTAL** | **62** | **57** |  |  | **48** |  |
