# Supplementary material for: Using hyperspectral leaf reflectance to estimate photosynthetic capacity and nitrogen content across eastern cottonwood and hybrid poplar taxa
Source: PLoS One. 2022 Mar 10;17(3):e0264780. doi: 10.1371/journal.pone.0264780 (PMC8912144; doi:10.1371/journal.pone.0264780)
Supplement: S2 Table — (DOCX) [file pone.0264780.s003.docx]

**S2 Table. Study objectives of selected vegetation indices.**

| **Source** | **Purpose of Study** |
| --- | --- |
|  |  |
| Carter et al. [104] | Detecting plant stress in persimmon, loblolly pine, slash pine, switchcane, golden euonymus, and live oak |
| Datt [99] | Leaf-level chlorophyll and carotenoid content estimation in Eucalyptus |
| Gamon [105] | Photosynthetic radiation use efficiency in top-canopy leaves of 20 different species |
| Gitelson and Merzlyak [106] | Leaf-level chlorophyll content estimation in horse chestnut and Norway maple |
| Gitelson and Merzlyak [107] | Autumn senescence and leaf-level chlorophyll content estimation in horse chestnut and Norway maple |
| Gitelson and Merzlyak [108] | Leaf-level chlorophyll content in horse chestnut, tobacco, vine, fig, Norway maple and catoneaster |
| Gitelson et al. [109] | Leaf-level chlorophyll content estimation in horse chestnut and Norway maple |
| Gitelson et al. [100] | Leaf-level chlorophyll content estimation in beech, elm, and wild vine |
| Gupta et al. [103] | Finding the relationship between red edge indices and leaf area index in wheat crop |
| Le Maire et al. [101] | Leaf-level chlorophyll content estimation in different deciduous broad leaf tree species |
| Maccioni et al. [102] | Leaf-level chlorophyll content estimation in croton, spotted eleagnus, Japanese pittosporum, and Benjamin fig |
| Penuelas et al. [110] | Assessing carotenoids/chlorophyll a ratio in the leaves of maize, wheat, tomato, soybean, sunflower, sugar beet, common oak, boxelder maple, and a succulent plant |
| Raper and Varco [98] | Finding the correlation between canopy reflectance and growth parameters and nitrogen status in cotton |
| Sims and Gamon [111] | Leaf pigment content estimation in 53 different plant species |
| Stimson et al. [112] | Estimating leaf water content and leaf water potential in Colorado pinon pine and one-seed juniper |
| Vogelmann et al. [113] | Leaf-level chlorophyll content estimation in sugar maple |
| Wen et al. [114] | Assessing nitrogen remobilization status in maize cultivars |
| Zarco-Tejada et al. [115] | Canopy-level chlorophyll content estimation in sugar maple |
