# Supplementary material for: Using hyperspectral leaf reflectance to estimate photosynthetic capacity and nitrogen content across eastern cottonwood and hybrid poplar taxa
Source: PLoS One. 2022 Mar 10;17(3):e0264780. doi: 10.1371/journal.pone.0264780 (PMC8912144; doi:10.1371/journal.pone.0264780)
Supplement: Attachment — Submitted filename: Response to Reviewers.docx [file pone.0264780.s004.docx]

**Responses to Reviewers’ Comments**

**Journal Requirements:**

1. When submitting your revision, we need you to address these additional requirements.

Please ensure that your manuscript meets PLOS ONE's style requirements, including those for file naming. The PLOS ONE style templates can be found at https://journals.plos.org/plosone/s/file?id=wjVg/PLOSOne_formatting_sample_main_body.pdf	and

https://journals.plos.org/plosone/s/file?id=ba62/PLOSOne_formatting_sample_title_authors_affiliations.pdf.

Based on the PLOS ONE formatting and file naming guidelines, we revised the titles of figures and tables as well as authors affiliations. We also uploaded the revised versions of Figs 1-4.docx and supporting information (S1 Fig and S1 & S2 Tables).docx in which we revised the titles based on PLOS ONE guidelines.

2. Please include your tables as part of your main manuscript and remove the individual files. Please note that supplementary tables (should remain/ be uploaded) as separate "supporting information" files.

In this revised manuscript, we included the tables in the main manuscript and deleted the individual files. We uploaded the supplementary tables and figure as a separate “supporting information” file.

3. Thank you for stating in your Funding Statement:

(This material is based upon work that is supported by the National Institute of Food and Agriculture, U.S. Department of Agriculture under award numbers 2018-67020-27934 to HJR and CMS, and 2018-68005-27636 to HJR and CMS, as well as U.S. Department of Agriculture McIntire Stennis Program under accession numbers: MISZ-067050 to HJR, MISZ-032100 to CMS, and MISZ-0621210 to KPP.

The funders had no role in study design, data collection and analysis, decision to publish, or preparation of the manuscript.)

Please provide an amended statement that declares *all* the funding or sources of support (whether external or internal to your organization) received during this study, as detailed online in our guide for authors at http://journals.plos.org/plosone/s/submit-now. Please also include the statement “There was no additional external funding received for this study.” in your updated Funding Statement.

Please include your amended Funding Statement within your cover letter. We will change the online submission form on your behalf.

We revised our Funding Statement in the cover letter. Thank you for doing the work for us. Our amended statement is as follows:

“This work was supported by the National Institute of Food and Agriculture, U.S. Department of Agriculture under award numbers 2018-67020-27934 to HJR and CMS, and 2018-68005-27636 to HJR and CMS (https://portal.nifa.usda.gov/lmd4/recent_awards), as well as U.S. Department of Agriculture McIntire Stennis Program under accession numbers: MISZ-067050 to HJR, MISZ-032100 to CMS, and MISZ-0621210 to KPP, respectively. The funders had no role in study design, data collection and analysis, decision to publish, or preparation of the manuscript. There was no additional external funding received for this study.”

4. We note that you have indicated that data from this study are available upon request. PLOS only allows data to be available upon request if there are legal or ethical restrictions on sharing data publicly. For more information on unacceptable data access restrictions, please see http://journals.plos.org/plosone/s/data-availability#loc-unacceptable-data-access-restrictions.

In your revised cover letter, please address the following prompts:

a) If there are ethical or legal restrictions on sharing a de-identified data set, please explain them in detail (e.g., data contain potentially sensitive information, data are owned by a third-party organization, etc.) and who has imposed them (e.g., an ethics committee). Please also provide contact information for a data access committee, ethics committee, or other institutional body to which data requests may be sent.

b) If there are no restrictions, please upload the minimal anonymized data set necessary to replicate your study findings as either Supporting Information files or to a stable, public repository and provide us with the relevant URLs, DOIs, or accession numbers. For a list of acceptable repositories, please see http://journals.plos.org/plosone/s/data-availability#loc-recommended-repositories.

We will update your Data Availability statement on your behalf to reflect the information you provide.

We uploaded our data at Scholars Junction: Mississippi State University’s Institutional Repository. In the cover letter, we revised our Data Availability statement and provided the link to access the data as follows:

“Our data can be accessed from Scholars Junction: Mississippi State University’s Institutional Repository at the following DOI: <https://doi.org/10.54718/BACR5952>.”

5. Please include your tables as part of your main manuscript and remove the individual files. Please note that supplementary tables (should remain/ be uploaded) as separate "supporting information" files.

In this revised manuscript, we included the tables in the main manuscript and deleted the individual files. We uploaded the supplementary tables and figure as a separate “supporting information” file.

**Additional Editor Comments:**

Both reviewers agree that this is a potentially a valuable contribution. However, they raise a number of important points that need to be addressed by the authors before the manuscript can be recommended for publication.

This revised manuscript addresses the concerns of the reviewers, and we also answer the questions of each reviewer. We made a substantial revision in this manuscript. Our major revisions are summarized as follows:

1) With a minor edit, we revised the title of our manuscript as “Using hyperspectral leaf reflectance to estimate photosynthetic capacity and nitrogen content across eastern cottonwood and hybrid poplar taxa”.
2) We revised the Introduction section.
3) We included the Tables in the main manuscript.
4) We provided the link to access our data and updated the Funding Statement in the cover letter.
5) We clarified the sample size used for (1) comparing taxa groups, (2) calculating the correlation coefficient matrix among parameters, and (3) developing LASSO and PCA models.
6) We described in more detail the methods we used for leaf sample selection, photosynthetic A/C_i_ curves measurements, hyperspectral leaf reflectance measurements, and statistical analysis.
7) In the Discussion section, we (1) justified why we used leaf N content on a per unit area basis (*N*_area_) instead of leaf N content on a per unit mass (*N*_mass_) basis; (2) justified why we decided to include the *N*_area_ model; (3) discussed the strengths and weaknesses of LASSO and PCA model development methods in its own paragraph; (4) merged the paragraphs with the same content while interpreting the hyperspectral wavelengths, and (5) provided more information about the significance of 935 nm

**Reviewers' comments:**

Reviewer's Responses to Questions

Comments to the Author

1. Is the manuscript technically sound, and do the data support the conclusions?

The manuscript must describe a technically sound piece of scientific research with data that supports the conclusions. Experiments must have been conducted rigorously, with appropriate controls, replication, and sample sizes. The conclusions must be drawn appropriately based on the data presented.

Reviewer #1: Yes

Reviewer #2: Yes

2. Has the statistical analysis been performed appropriately and rigorously?

Reviewer #1: Yes

Reviewer #2: Yes

3. Have the authors made all data underlying the findings in their manuscript fully available?

The PLOS Data policy requires authors to make all data underlying the findings described in their manuscript fully available without restriction, with rare exception (please refer to the Data Availability Statement in the manuscript PDF file). The data should be provided as part of the manuscript or its supporting information, or deposited to a public repository. For example, in addition to summary statistics, the data points behind means, medians and variance measures should be available. If there are restrictions on publicly sharing data—e.g. participant privacy or use of data from a third party—those must be specified.

Reviewer #1: Yes

Reviewer #2: Yes

4. Is the manuscript presented in an intelligible fashion and written in standard English?

PLOS ONE does not copyedit accepted manuscripts, so the language in submitted articles must be clear, correct, and unambiguous. Any typographical or grammatical errors should be corrected at revision, so please note any specific errors here.

Reviewer #1: Yes

Reviewer #2: Yes

**5. Review Comments to the Author**

Please use the space provided to explain your answers to the questions above. You may also include additional comments for the author, including concerns about dual publication, research ethics, or publication ethics. (Please upload your review as an attachment if it exceeds 20,000 characters)

**Reviewer #1:** The authors proposed using hyperspectral leaf reflectance to estimate the photosynthetic capacity and nitrogen content, and modeled it by LASSO and PCA, so as to verify that hyperspectral reflectance modeling can be used as a cost-effective means to conduct extensive field phenotyping to quickly screen *Populus* genotypes. The language is fluent and there are few small mistakes. The methods are innovative, with strong innovation, reliable experimental design and detailed data, however, there are some problems, such as the lack of organization of each part and disorderly writing. The manuscript should be substantial revisions.

Thank you for providing helpful comments. Following your suggestions, we made a significant amount of revisions and improved the structure of each section so as to achieve more organized writing.

**Introduction**

The second paragraph (Line 65), is necessary to strengthen the importance of "photosynthetic parameters" and "leaf nitrogen content".

We added additional information about the importance of photosynthetic capacity parameters and leaf nitrogen content and their significant relationship with biomass productivity with the following additions:

“To this end, photosynthetic capacity can be used as a selection criterion for clonal screening because individuals with enhanced photosynthetic rate under field conditions are likely to result in higher biomass yield [30].” (Lines 72 – 74)

“In addition to photosynthetic capacity parameters, nutrient content in leaves is another important parameter for plant growth because limitations of nutrients can decrease photosynthesis.” (Lines 79 – 81)

“Additionally, leaf N content tends to be linearly correlated with photosynthetic capacity [37, 38], and consequently, variations in leaf N content can affect biomass production [39-41].” (Lines 82 – 84)

The third paragraph (Line 87) needs to introduce the relationship between spectral data and "photosynthetic parameters", "leaf nitrogen content per unit area". For example, how does the spectral data behave when the net photosynthetic rate is high or leaf nitrogen is high? And clarify the advantages and disadvantages of the field experiments and hyperspectral leaf reflectance.

In this revision, we introduced the relationship between leaf spectra, photosynthetic capacity parameters, and leaf nitrogen content. We included relevant literature on which parts of electromagnetic spectrum were sensitive to increases or decreases in photosynthetic rates and leaf nitrogen content (Lines 93 – 96 and 97 – 99). We also provided the strengths and weaknesses of each method (Lines 84 – 89 and 97 – 102). We added the following information in this revised manuscript:

“Therefore, there is a physiological relationship between leaf reflectance and photosynthetic capacity parameters [43-45]. For example, according to Qian et al. [44], when *V*_cmax_ increases, the reflectance at the red edge position and the green peak decreases.” (Lines 93 – 96)

“Ye et al. [47] found that when leaf N content increased, spectral reflectance decreased, and this relationship was more significantly observed in the green to near infrared regions.” (Lines 97 – 99)

“On the other hand, creating empirical models for hyperspectral data requires sophisticated model development techniques, and model robustness depends on the quality of sample data measured in the field [53].” (Lines 102 – 104)

The fourth paragraph (Line 104) puts forward many methods for analyzing hyperspectral data. Why did the author choose LASSO and PCA? To the best of my knowledge, the stepwise regression of multiple linear regression and ridge regression are also good methods to deal with multicollinearity. What are the advantages and disadvantages of the two models selected? These should be described in the article.

We justified why LASSO and PCA methods were more optimal than stepwise and ridge regressions for analyzing hyperspectral data. In our data, the number of predictor variables (*P* = 512) was significantly larger than sample size (*n* = 105). Under this small-*n*-large-*P* condition, stepwise regression of multiple linear regression is not an optimal option due to its possible selection of nuisance variables. Ridge regression selects all predictor variables in its final model, and our data have 512 predictor variables. Consequently, ridge regression has drawbacks in building model for our hyperspectral data. We also provided explanations about why LASSO and PCA can perform better than other methods. We added the following information in this revised manuscript:

“Hyperspectral data also contain hundreds of spectral bands, and therefore hundreds of predictor variables. If the number of predictor variables is greater than the sample size, stepwise selection of multiple linear regression likely chooses nuisance variables rather than true variables, and consequently, performs poorly for out-of-sample accuracy [59, 60]. Ridge regression includes all predictor variables in the final model, which is disadvantageous when analyzing data containing a large number of predictor variables [59]. To solve these issues, many studies suggested LASSO [61-63] and PCA [64-66] methods. The PCA method is easy and computationally efficient. Its major drawback is a difficulty in interpreting principal components because they represent linear combinations of the original features, and thus, each component uses the collection of all predictor variables [67]. LASSO can overcome this limitation of PCA. Compared to PCA, LASSO is likely to generate a simpler model because LASSO uses regularization, shrinkage, and variable selection, and the final model includes a smaller set of predictor variables [59]. However, due to being a supervised feature extraction method [68-70], LASSO is more computationally demanding than PCA.” (Lines 114 – 127)

The last paragraph (Line 123) of Introduction should add some introduction concerning the importance of poplar in Mississippi, such as planting area, age of forest, and annual yield and so on.

We added more information about *Populus*, such as optimal harvesting cycle, annual biomass productivity of *Populus* genotypes, and its potential as a bioenergy crop classified by the United States Department of Energy. Rather than in the last paragraph, we described this information in the first paragraph, which was the section talking about bioenergy and *Populus* genotypes.

Please note that planting *Populus* for producing biomass feedstocks production is in its beginning stages and there is not large-scale, commercial planting at this time. We did this study not because *Populus* is widespread across Mississippi and the USA, but because it has the potential to become a promising bioenergy crop. We described about this with the following additions:

“Currently in the USA, *Populus* feedstock production for bioenergy makes up a small portion of land use, but there is the potential for large-scale plantations for bioenergy in the future because *Populus* is a promising bioenergy crop.” (Lines 53 – 56)

“The biomass yield of *Populus* genotypes can reach 7.5 to 15.2 Mg/ha/year [13]. Due to their rapid growth and high biomass productivity, the United States Department of Energy classifies *Populus* as a potential SRWC for bioenergy production [14]. A *Populus* plantation can be managed with a five-year harvesting cycle [10, 15-17], and ...” (Lines 58 – 61)

**Materials and Methods**

Line 164, photosynthetic traits measurement should be described more detailed, measurement time and conditions should be clarified. See Liu et al. (2021).

Liu X, Zhang Q, Song M, Wang N, Fan P, Wu P, Cui K, Zheng P, Du N, Wang H, and Wang R. Physiological response of Robinia pseudoacacia and Quercus acutissima seedlings to repeated drought-rewatering under different planting methods. Frontiers in Plant Science; 2021; 12:760510.

We provided more details about our leaf sampling protocol, number of leaves we measured in each taxa group, measurement time, and conditions, such as photosynthetic photo flux density, leaf temperature, and relative humidity concentration inside the leaf chamber during gas exchange measurement (Lines 172 – 207).

Line 224, why this study used leaf N content on a per unit area instead of leaf N content on a per unit mass?

Initially, we analyzed both leaf N content on a per unit area (*N*_area_; g N/m^2^) and leaf N content on a per unit mass (i.e., *N*_mass_ (%); calculated by dividing leaf N mass with leaf sample weight and multiplying with 100). However, the model of *N*_area_ fit the data better and had higher *R*^2^ than that of *N*_mass_ (%). So, we reported only the results of *N*_area_ model and excluded the leaf N mass % model. In this revised manuscript, we explained about this with the following addition:

“In addition to *N*_area_, we also developed a model for leaf N content per unit mass (i.e., *N*_mass_ (%); calculated by dividing leaf N mass with leaf sample mass and then, multiplying with 100). Nonetheless, the *N*_area_ model (*R*^2^ = 0.29 for LASSO and 0.15 for PCA) fit the data better than that of *N*_mass_ (%) (*R*^2^ = 0.07 for LASSO and 0.02 for PCA). *N*_area_ resulted in a better model likely because *N*_area_ incorporated the information of both *N*_mass_ and leaf mass per area in its calculation.” (Lines 402 – 407)

**Discussion**

There are too many paragraphs in Discussion, and the meaning of some paragraphs is unclear, for example, what is the significance of the interpretation of the wavelength at 935 nm (Line 423)? It is necessary to sort out the structure again. The paragraphs on Line 452 and 466 are the discussion of leaf nitrogen content, which can be combined.

It is suggested to separate the Discussion in some parts including, (1) the relationship between traits and spectral data, (2) the discussion of LASSO model, (3) the discussion of PCA model, (4) a comparison among the two models and any other models. The parts above are for your reference only, but they are not appropriate as subtitles. The authors may either divide the discussion into several parts according to the content; or merges paragraphs with similar content.

Thank you for the helpful comment on how to restructure the Discussion. While we did not use this exact order, we thoroughly revised the Discussion section to improve the flow and readability. We still kept the Discussion into two main sections: “Model performance” and “Interpretation of selected models”. However, in the “Model performance” section, we divided the discussion into five separate paragraphs: (1) performance of the models and justification about why the *N*_area_ model had lower *R*^2^ and why we used leaf N content on a per unit area basis instead of leaf N content on a per unit mass basis (Lines 395 – 407), (2) using a diverse range of taxa and genotypes to represent genetic variability while developing the models (Lines 408 – 418), (3) discussion about LASSO model (Lines 419 – 434), (3) discussion about PCA model (Lines 435 – 449), and (4) comparison with published vegetation indices (Lines 450 – 466). We also discussed the pros and cons of LASSO model vs. PCA model in the third and fourth paragraphs (Lines 419 – 449).

In the “Interpretation of selected wavelengths” section, we merged the paragraphs into similar content, such as (1) the two paragraphs of *V*_cmax_ and *J*_max_ wavelengths were combined into one (Lines 482 – 515), which described the relationship between traits and spectral wavelengths: 758 and 935 nm, and (2) the two paragraphs of leaf nitrogen content were combined into one (Lines 532 – 561). We also added more information about the significance of 935 nm and its relationship with photosynthetic capacity (Lines 498 – 515).

**Reviewer #2:** The manuscript reports a study that estimated the photosynthetic capacity and nitrogen content of eastern cottonwood and hybrid Poplar genotypes using hyperspectral leaf reflectance. The results help quickly screen Poplar genotypes with maximum yield potential. However, the manuscript has some significant flaws with the method description. A major revision should be conducted before considering for publication in PLOS ONE. Key concerns with the manuscript are summarised below.

1, What are the soil physicochemical properties (i.e., total N content, organic matter) in those study areas, i.e., Monroe County, Pontotoc County;

We added the information about soil properties of our two study sites, such as soil carbon, soil nitrogen, organic matter, and dry bulk density at the Monroe (Lines 159 – 161) and Pontotoc (Lines 166 – 168) sites as follows:

“This site has soil carbon, soil nitrogen, organic matter, and dry bulk density of 2.29 kg/m^2^, 0.21 kg/m^2^, 1.08%, and 1.60 g/cm^3^, respectively.” (Lines 159 – 161)

“This site has soil carbon, soil nitrogen, organic matter, and dry bulk density of 3.61 kg/m^2^, 0.44 kg/m^2^, 1.83%, and 1.52 g/cm^3^, respectively.” (Lines 166 – 168)

2, The authors need to provide more detailed information about the gas exchange measurement, i.e., leaf position in the branches, were the selected leaves fully expanded?

We provided more detailed information about our gas exchange measurement, such as measurement time, place, and conditions (including photosynthetic photo flux density, leaf temperature, and relative humidity concentration inside the leaf chamber during gas exchange measurement), leaf selection and sampling protocol, etc. (Lines 172 – 194).

3, How many times did the authors determine the photosynthetic parameters in mid-July and early September in 2019 at the Monroe site and mid-July 2020 at the Pontotoc site? As we know, every time the photosynthetic parameters of those 62 genotypes were determined, the measurement should be conducted at a same time or during the same period, in order to make the comparison between different genotypes reasonable, however, the mid-July and early September in 2019 were a long period.

We added measurement dates in S1 Table. We measured a total of 105 leaves (*n* = 105). Among them, at the Pontotoc site, we measured 48 leaves from trees in their second growing season in 2020 (16 leaves on July 14, 16 leaves on July 15, and 16 leaves on July 16). At the Monroe site, we measured 57 leaves from trees in their second growing season in 2019 (10 leaves on July 18, 4 leaves on July 25, 13 leaves on July 26, 10 leaves on September 2, and 6 leaves on September 4). Therefore, we measured 85 % of leaf samples in July and 15% in September, and September measurements were re-measurements of formerly measured genotypes in July. We described about these in this revised manuscript as follows:

“In total, about 85% of leaf samples were measured in July and 15% in early September. September measurements were made on previously measured genotypes from July because some spectral response curves from leaves measured in July were more variable than the majority of collected data, and thus, these genotypes were re-measured.” (Lines 197 – 200)

In this revision, we also clarified that we made a statistical comparison for the taxa level (not at the genotype level) at Lines 259 – 268.

4, How many repetitions of the photosynthetic parameters measurement did the authors conducted for each genotypes? According to the description in the manuscript, the repetitions of the photosynthetic parameters measurement for each genotypes seemed less than 3, which was not sufficient for the statistical analysis.

We did not make statistical comparison among genotypes. We made comparisons at the taxa level. Therefore, we combined the leaf sample data based on taxa and performed Kruskal-Wallis test (Lines 259 – 268). The number of leaf samples we measured for each taxa group were 27 leaves for D×D, 27 for D×M, 31 for D×N, 13 for D×T, and 7 for T×M, respectively (Note: we added T×D genotype into D×T, and (D×N)×M genotype into D×N due to their small sample size) (Lines 264 – 271). We did not have any leaf samples measured for (D×N)×M and T×M at the Monroe site. In S1 Table, we described the number of leaves we measured for each taxa group at the Monroe and Pontotoc sites.

To develop LASSO and the PCA models (Lines 274 – 276 and 299 – 300) and to establish the correlations among parameters (Lines 269 – 271), we used all the measured leaf sample data (*n* = 105), and we did not discriminate or group based on taxa or genotypes for these.

5, In the Fig.1, Fig.3, Table 1 and Table, how many values for each parameter of each *Populus* genotype were used for the statistical analyses, i.e., *n*=?

In S1 Table, we described the number of leaves we measured for each taxa and genotype at the Monroe and Pontotoc sites.

Fig 1 showed the statistical differences among taxa group. We had five taxa groups: D×D, D×M, D×N, D×T, and T×M. Since we made statistical comparison at the taxa-level (not at the genotype-level), we pooled the data based on taxa and performed the Kruskal-Wallis test for taxa comparison. We measured the physicochemical parameters from a total of 105 leaves, and the number of leaf samples we measured for each taxa group were 27 leaves for D×D, 27 for D×M, 31 for D×N, 13 for D×T, and 7 for T×M, respectively.

Fig 3 showed the scatterplots of LASSO and PCA models for each parameter. Table 1 showed the correlation among the physicochemical parameters. Table 2 showed LASSO and PCA models and their model parameters. For Fig 3, Table 1, and Table 2 results, we did not group the measured data based on genotypes or taxa. Instead, we used all leaf sample data (i.e., *n* = 105) measured across *Populus* taxa and genotypes to develop the models and to calculate a Pearson correlation coefficient matrix among parameters. The model we developed was not intended for either taxa- or genotype-specific predictions. Therefore, we used all leaf sample data measured across all taxa and genotypes during development of these models.

In this revised manuscript, we also clarified about the sample size (*n*) we used for (1) taxa comparisons (Lines 259 – 268), (2) correlations across parameters (Lines 269 – 271), and (3) model development (Lines 274 – 276 and 299 – 300).

6, The leaf spectra has been successfully used for representing the leaf N content, but in this research, why the *R*^2^ of the LASSO or PCA model for *N*_area_ was the lowest compared with that of *V*_cmax_, *J*_max_ and TPU (Table 3)? The value was only 0.29 or 0.15, it seemed that the leaf spectral reflectance showed weak correlation with N conten.

We were aware of the fact that the *N*_area_ model had lower *R*^2^ (0.29 for LASSO and 0.15 for PCA) compared to photosynthetic capacity parameters, which had *R*^2^ of 0.50 to 0.56. The possible reason was that *N*_area_ was also found to be weakly correlated with photosynthetic data suggesting that a large portion of N in leaves was not involved in photosynthesis (Table 1), and therefore, did not generate a significant spectral signal. However, we decided to include the results of *N*_area_ model because of these two reasons:

(1) The root mean square percentage error (RMSPE; calculated as RMSE/$\overline{X}$) of *N*_area_ model (21% for LASSO model and 23% for PCA model) was lower than that of photosynthetic capacity parameters, which had 31 to 33% (Table 2).
(2) We also found that all four wavelengths selected in the *N*_area_ model were previously reported as wavelengths, which were significantly associated with leaf N content.

We also described this in the Discussion section (Lines 396 – 402 and 532 – 537).

7, The hyperspectral leaf reflectance would be easily influenced by the leaf growth status and its surroundings, were all the parameters determined at the same time and in the same conditions, if not, how did the authors ensure that the leaf reflectance varied just as the photosynthetic parameters changed? And was the establishment of the relationship between these parameters reasonable?

We used the same leaves for measuring both photosynthetic A/C_i_ response curves and hyperspectral leaf reflectance. We paired measurements of these two parameters together in close proximity temporally and under the same environmental and light conditions at both study sites. Immediately after acquiring the photosynthetic A/C_i_ response curves, we completed the leaf reflectance measurements. Under saturating light conditions at 1500 µmol/m^2^/s inside the leaf chamber of LI-COR 6400 XTP, gas exchanges of the sample leaves were measured on the sunlit side of trees. Within a few minutes of photosynthetic measurements, we measured the hyperspectral leaf reflectance while it was sunny. All measurements were conducted between 10:00 AM and 2:00 PM on clear sunny days to ensure that there was abundant sunlight during measurement. Therefore, we measured both photosynthetic capacity parameters and leaf reflectance at about the same time and in the same conditions because there was a few minutes of time gap for setting up the shift of measurements. Consequently, developing the relationship between these parameters was reasonable. We also mentioned about this in this revised manuscript as follows:

“Immediately after gas exchange measurements of a leaf, hyperspectral leaf reflectance was measured on the same leaf so that both measurements were made in close proximity temporally and under the same environmental and light conditions.” (Lines 213 – 215)

8, The authors intended to establish methods for rapidly estimating those physicochemical parameters of different *Populus* genotypes, and quickly screening genotypes with maximum yield potential. In this research, there were 62 *Populus* genotypes, maybe there should be 62 group data of photosynthetic parameters, leaf N content and hyperspectral leaf reflectance values, respectively. However, only 7 group data were showed in Figure 1. Did the authors establish the correlations between these two parameters by using these 7 group data or 62 group data?

We used all leaf samples (*n* = 105) measured across 7 taxa and 62 genotypes of *Populus* to establish the correlations among parameters (Lines 269 – 271). For this correlation establishment, we did not group the measured data based on taxa or genotypes.

In Fig 1, we showed the box plots of physiochemical parameters for 7 different *Populus* taxa. In this case, we wanted to report the statistical differences among taxa. Since this comparison was made at the taxa-level (not the genotype-level comparison), we grouped the data based on taxa and performed the Kruskal-Wallis test for taxa comparison (Lines 259 – 268).

While developing LASSO or PCA models, we used all leaf samples (*n* = 105) (Lines 274 – 276 and 299 – 300). The models we developed were not intended for either taxa- or genotype-specific predictions. We also explained why developing a taxa- or genotype-specific model is not suggested for clonal screening with the following addition:

“For screening, new genotypes are developed by breeding within and potentially between taxa. Therefore, creating models from taxa- or genotype-specific data may not be robust enough because new genotypes that need to be screened for productivity potential are being developed.” (Lines 408 – 410)
